# Comparative analysis of the efficacy of a transverse process bone graft with other bone grafts in the treatment of single-segment thoracic spinal tuberculosis

**DOI:** 10.1186/s13018-019-1312-9

**Published:** 2019-09-02

**Authors:** Zhongyuan He, Ke Tang, Fei Gui, Yuan Zhang, Weiyang Zhong, Zhengxue Quan

**Affiliations:** 1 Department of Orthopedics, The First Affiliated Hospital of Chongqing Medical University, Yuanjiagang, Yuzhong District, Chongqing, 400016 China; 2 Department of Orthopedics, University-Town Hospital of Chongqing Medical University, Shapingba District, Chongqing, 401331 China; 3 Department of Orthopedics, Children’s Hospital of Chongqing Medical University, Yuzhong District, Chongqing, 401122 China

**Keywords:** Single-segment, Thoracic spinal tuberculosis, Posterior debridement, Transverse bone graft, Comparison

## Abstract

**Background:**

There was a controversy about bone grafting of spinal tuberculosis treatment. The aim of this study was to compare the clinical efficacy of a new bone grafting method—transverse bone grafting (TBG)—with iliac bone grafting (IBG) and titanium mesh grafting (TMG) in the treatment of single-segment thoracic spinal tuberculosis.

**Material and methods:**

TBG was undertaken in 30 patients (group A), IBG was carried out in 28 patients (group B), and TMG was performed in 36 patients (group C). The operative time, intraoperative blood loss, postoperative drainage amount, postoperative complications, length of hospital stay, erythrocyte sedimentation rate (ESR), C-reactive protein (CRP) level, visual analog scale (VAS) score for back pain, Oswestry dysfunction index (ODI), intervertebral height, and time to bone graft fusion were compared. Changes in the Cobb angle of kyphosis, intervertebral height, and loss to the final follow-up were measured. Neurological function recovery was evaluated according to the criteria of the American Spinal Injury Association (ASIA).

**Results:**

The operative times in group A was significantly shorter than those in groups B and C (*P*_AB_ = 0.036, *P*_AC_ = 0.005, *P*_BC_ = 0.901). The hospital stay in group A was significantly shorter than that in groups B and C (*P*_AB_ = 0.022, *P*_AC_ = 0.031, *P*_BC_ = 0.424). The intraoperative blood loss in group A was significantly less than that in groups B and C (*P*_AB_ = 0.045, *P*_AC_ = 0.004, *P*_BC_ = 0.586). The VAS score, ODI, ESR level, CRP level, Cobb angle of kyphosis, and intervertebral height of the affected segment were significantly improved compared with those before surgery (*P* < 0.05).

**Conclusion:**

For the treatment of single-segment thoracic spinal tuberculosis, the new interbody fusion technique using transverse process bone grafting is a safe, reliable, effective, and ideal bone grafting method.

## Background

Spinal tuberculosis is a chronic granulomatous disease caused by *Mycobacterium tuberculosis* infection and is often secondary to lung tuberculosis. The thoracic and lumbar vertebrae are commonly affected sites. As the most common form of extrapulmonary tuberculosis, spinal tuberculosis has an incidence of approximately 2–3% [1, 2]. Vertebral body collapse is associated with bone destruction due to tuberculosis, which leads to kyphosis deformity, neurological damage, spinal cord compression, and even paraplegia in severe cases, thus affecting the health of the population [3].

Effective antituberculosis treatment, complete lesion removal, and secure fusion of implants are considered the three major elements for successful treatment of thoracolumbar tuberculosis [4]. Among them, bone graft fusion plays important roles in bone defect repair after the removal of a tuberculosis lesion, correction of kyphosis, and reconstruction of spinal stability [5, 6].

Currently, the bone graft materials commonly used in the surgical treatment of thoracic tuberculosis include autologous iliac crest, particulate bone graft, allogeneic bone graft, adjacent rib graft, and titanium mesh with bone powders, while transverse process bone grafts have rarely been reported. Traditional grafting with an iliac bone block, adjacent rib graft, and titanium mesh with bone powders is associated with a long operative time, massive intraoperative blood loss, and a slow postoperative recovery. A particulate bone graft has a loose structure that cannot provide sufficient mechanical strength [7]. To address this issue, we sought a new bone grafting method to minimize donor site complications, ensure sufficient support strength and a satisfactory fusion rate, and help patients achieve a quick recovery after surgery.

The authors analyzed 94 patients with single-segment thoracic tuberculosis who were treated in the First Affiliated Hospital of Chongqing Medical University from January 2013 to September 2017 via a posterior-only approach debridement, internal fixation. This study explored the clinical efficacy and differences between different types of bone grafts in the treatment of single-segment thoracic spinal tuberculosis. We report the results as follows.

## Material and methods

### General data

We conducted a retrospective analysis of 94 patients with single-segment thoracic tuberculosis who met the inclusion criteria for spinal surgery from January 2013 to September 2017 in our hospital. The selected patients were divided into three groups according to the type of bone graft: group A, transverse process bone graft fusion and internal fixation (30 patients); group B, autologous iliac bone graft and internal fixation (28 patients); and group C, titanium mesh fusion and internal fixation (36 patients). No significant differences in age, gender, body mass index, or disease duration were found between the four groups (*P* > 0.05, Tables 1, 2, and 3).

**Table 1:** Comparison of preoperative clinical features between the three groups

Group	Case	Age (year)	Gender		BMI	Course of illness (month)
			Male	Female		
Transverse bone (A)	30	44.33 ± 14.16	20	10	21.22 ± 2.91	18.03
Iliac bone graft (B)	28	46.32 ± 14.07	12	16	22.09 ± 3.74	24.50
Titanium mesh (C)	36	49.97 ± 16.36	21	15	20.97 ± 3.08	26.00
*P* value		>0.05	> 0.05		>0.05	> 0.05

**Table 2:** Comparison of clinical features between the three groups (*x* ± *s*)

	Group A	Group B	Group C	*P*_AB_	*P*_AC_	*P*_BC_
Fusion time (m)	7.3 ± 2.1	8.4 ± 3.1	7.5 ± 3.4	0.642	0.101	0.616
Hospital stay (days)	18.8 ± 5.1	24.0 ± 12.7	26.1 ± 9.5	0.022	0.031	0.424
Operation time (min)	205.5 ± 38.1	240.7 ± 68.4	238.4 ± 45.4	0.036	0.005	0.901
Operation blood loss (ml)	372.8 ± 151.1	480.9 ± 241.8	603.1 ± 443.2	0.045	0.004	0.586
Postoperative drainage (ml)	292.1 ± 126.0	381.0 ± 212.2	377.6 ± 139.0	0.715	0.672	0.998
Preoperation CRP (mg/l)	39.0 ± 32.2	31.7 ± 21.3	29.5 ± 27.83	0.459	0.661	0.111
Postoperation CRP (mg/l)	53.7 ± 31.8	52.1 ± 31.7	51.0 ± 30.1	0.667	0.456	0.382
Preoperation ESR (mm/h)	22.1 ± 17.9	14.4 ± 10.8	18.2 ± 16.2	0.247	0.375	0.460
Postoperation ESR (mm/h)	31.2 ± 24.7	31.7 ± 27.9	33.2 ± 19.1	0.701	0.503	0.196
Preoperation VAS score	6.1 ± 1.9	5.9 ± 1.7	5.9 ± 1.5	0.002	0.447	0.813
Follow-up end VAS score	1.4 ± 1.4	1.3 ± 0.9	1.3 ± 1.1	0.780	0.472	0.695
Preoperation ODI	55.0 ± 9.9	54.3 ± 15.2	57.9 ± 12.3	0.912	0.545	0.655
Follow-up end ODI	14.2 ± 6.5	15.7 ± 6.3	16.9 ± 8.2	0.682	0.125	0.265

*ESR* erythrocyte sedimentation rate, *CRP* C-reactive protein, *P* < 0.05 compared with preoperation

**Table 3:** Comparison of the imaging features between the three groups (*x* ± *s*)

	Group A	Group B	Group C	*P*_AB_	*P*_AC_	*P*_BC_
Preoperation Cobb angle (°)	25.3 ± 7.1	26.7 ± 5.6	29.1 ± 4.8	0.247	0.287	0.391
Postoperation Cobb angle (°)	15.5 ± 4.1	15.1 ± 2.4	14.5 ± 2.9	0.133	0.732	0.386
Follow-up end Cobb angle (°)	16.9 ± 4.4	15.5 ± 2.9	16.2 ± 4.0	0.248	0.725	0.486
Preoperative height (cm)	10.6 ± 1.8	11.1 ± 1.5	11.2 ± 1.6	0.972	0.799	0.879
Postoperative height (cm)	14.1 ± 1.3	13.9 ± 1.4	13.9 ± 1.5	0.195	0.985	0.907
Correction height (cm)	3.3 ± 0.8	2.9 ± 1.1	3.3 ± 1.8	0.467	0.413	0.585
Follow-up end height (cm)	12.6 ± 1.5	12.0 ± 1.5	11.9 ± 1.5	0.939	0.528	0.796
Loss height (cm)	1.5 ± 0.5	1.9 ± 1.1	1.9 ± 0.7	0.953	0.066	0.323

*P* < 0.05 compared with preoperation Cobb angle; *P* < 0.05 compared with postoperation Cobb angle

### Inclusion and exclusion criteria

The inclusion criteria for the patients in the three groups include the following: (1) patients with single-segment thoracic spinal tuberculosis (involvement of a single segment and a lesion requiring removal), varying degrees of vertebral destruction or collapse, and narrowing or disappearance of the intervertebral space as confirmed by preoperative X-ray, computed tomography (CT), and magnetic resonance imaging (MRI) examinations; (2) patients in good condition who can tolerate surgery; (3) patients with surgical indications who underwent posterior-approach bone graft fusion surgery alone and a diagnosis of tuberculosis confirmed by postoperative pathological examination; and (4) patients with complete follow-up data.

The exclusion criteria for the three groups of patients include the following: (1) patients complicated with severe heart and lung dysfunction or other chronic diseases who cannot tolerate surgery; (2) patients with incomplete clinical data; (3) patients with a history of thoracic spine fractures within 6 months and a previous history of thoracic spinal surgery; (4) patients with severe local kyphosis requiring osteotomy and orthosis; (5) patients with active tuberculosis and intestinal tuberculosis; and (6) patients with malignant tumors, mental illness, or hyperthyroidism.

### Preoperative preparation

All patients received antituberculosis therapy for 2 to 4 weeks before surgery (the oral chemotherapy regimen included rifampicin 450 mg/day, isoniazid 300 mg/day, pyrazinamide 1500 mg/day, and ethambutol 750 mg/day). Surgery was performed when the typical symptoms of tuberculosis were significantly relieved and the systemic condition of the patient was good.

### Surgical procedure

**Fig. 1:**
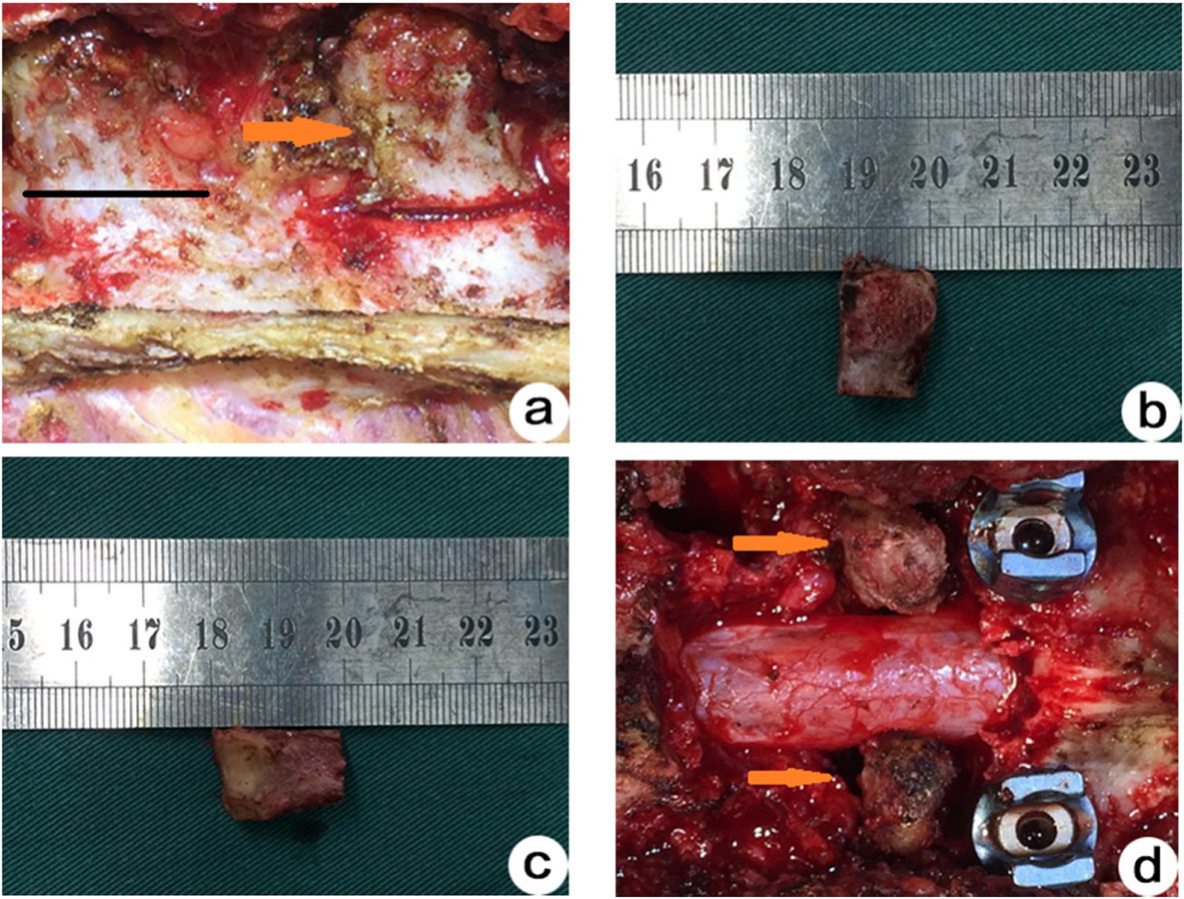
Transversus bone graft group: **a**-**c** A transverse process was harvested from a normal adjacent vertebra. Both ends of the transverse process were trimmed and ground to create a columnar cage with annular cortical bone on the sides and cancellous bone at both ends. **d** According to the size of the intervertebral space defect, one to two cages created from transverse processes were implanted. A pedicle screw system was used to immobilize bone grafts and vertebral segments

**Fig. 2:**
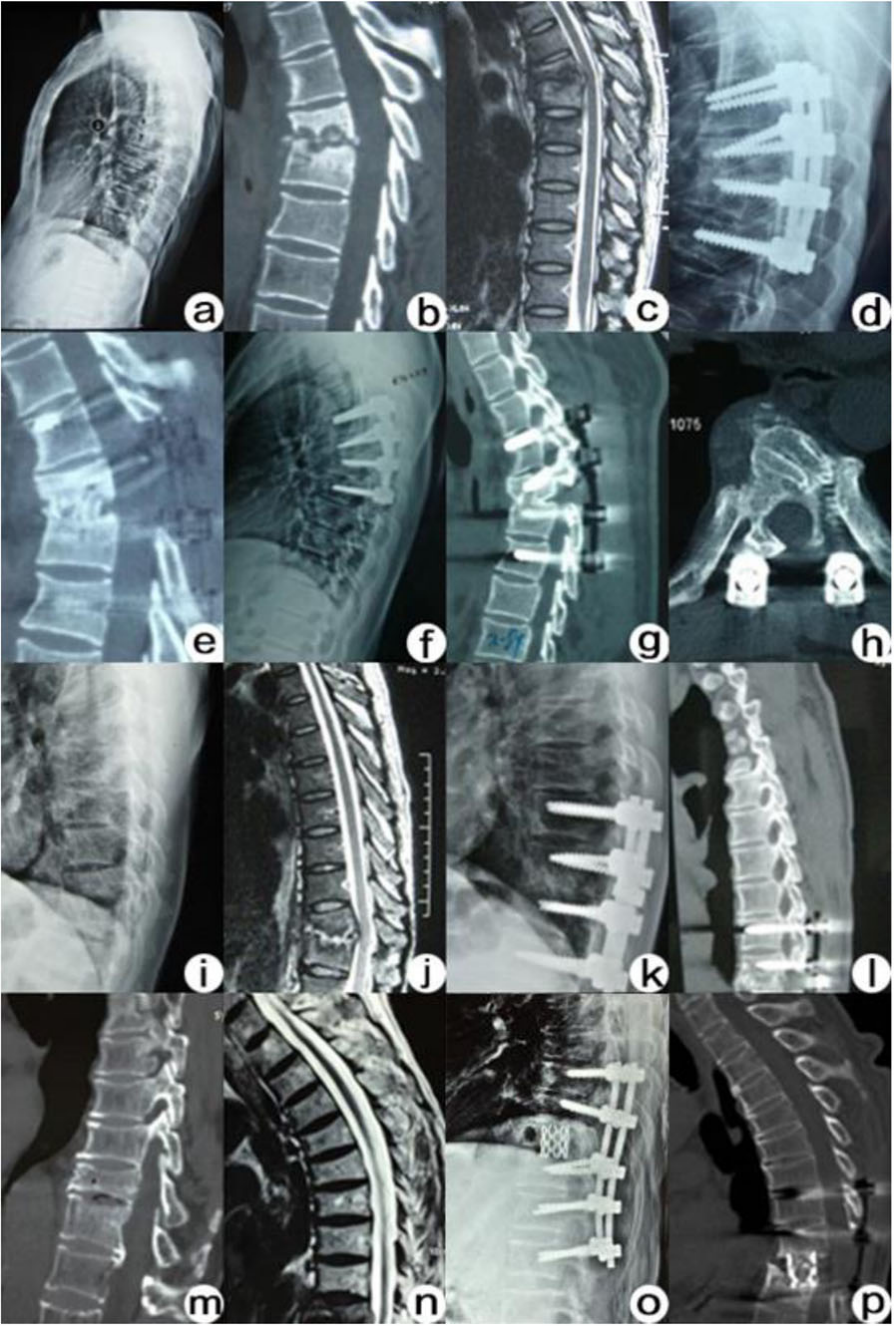
**a**-**h** A 40 years old male with T6-7 tuberculosis. **a**-**c** Preoperation X-ray, CT and MRI images showed irregular destruction of the edge of T6-7 vertebral body and intervertebral space stenosis; (**d**-**e**) Six months postoperation X-ray and CT showed good internal fixation position. **f**-**h** Thirty months postoperation X-ray, CT and MRI showed good internal fixation position. **i**-**l** Iliac bone graft group: a 41 years old male with T10-T11 tuberculosis (**i**-**j**). Preoperation X-ray and MRI images showed that T10-T11 vertebrae presented osteolytic bone destruction and intervertebral space stenosis; (**k**-**l**) twelve months postoperation X-ray and CT showed T10-T11 bone fusion. **m**-**p** Titanium cage graft group: a 69 years old male with T11-12 tuberculosis. **m**-**n** Preoperation CT and MRI images showed that T11-12 vertebrae presented osteolytic bone destruction and intervertebral space stenosis; (**o**-**p**) eighteen months postoperation X-ray and CT showed T11-T12 bone fusion

The patient was placed in the prone position after induction of general anesthesia with tracheal intubation. C-arm fluoroscopy was performed to identify the diseased vertebra. A posterior median incision was used to expose the spinous process, lamina, facet joint, and transverse process of the normal vertebral body above and below the diseased vertebra via subperiosteal dissection. A needle was inserted for localization of the diseased vertebra. C-arm fluoroscopy was used to confirm the diseased segments. Pedicle screws were placed in the one or two normal vertebrae above or below the diseased vertebra and in the diseased vertebrae if possible. The bilateral laminas of the diseased segment were removed to expose the dural sac and the nerve root. Spinal decompression was performed under direct vision. A connecting titanium rod with a suitable length and curve was installed. The lesion tissue (inflammatory granulation tissue, necrotic intervertebral disc, and lesioned bone) was completely scraped out until fresh oozing on the vertebrae was observed, and the lesion site and wounds were repeatedly washed with a large amount of normal saline. The intervertebral space was properly expanded to form a bed for the bone graft, and the different types of bone grafting were then performed for the designated groups (Figs. 1 and 2).

#### Group A (transverse process bone graft)

A transverse process was harvested from a normal adjacent vertebra. Both ends of the transverse process were trimmed and ground to create a columnar cage with annular cortical bone on the sides and cancellous bone at both ends (Fig. 1). According to the size of the intervertebral space defect, one to two cages created from transverse processes were implanted. A pedicle screw system was used to immobilize bone grafts and vertebral segments (Fig.1).

#### Group B (iliac bone graft)

Preoperative imaging results were used to estimate the size of the bone defect. The patient was placed in the supine position, and an appropriately sized autogenous iliac bone block was harvested. The block with cortical bone on three sides was trimmed to an appropriate size and implanted into the bone defect site by “inserting along its short axis and fixating along its long axis.” The internal fixation was adjusted by applying pressure for immobilization and fusion of the bone.

#### Group C (titanium mesh implant)

After determining the size of the intervertebral body defect, an appropriately sized titanium mesh (filled with bone powders from the normal lamina and articular processes removed during surgery) was implanted in the defect site. The pedicle screw system was adjusted with pressure to ensure immobilization of the graft.

After bone grafting, streptomycin (1.0 g) and isoniazid (300 mg) were placed in the lesion site, and two drainage tubes were placed in the incision. The incision was closed in layers, and the excised specimen was sent for bacterial culture and pathological examination.

### Postoperative management

The oral isoniazid, rifampicin, pyrazinamide, and ethambutol (HREZ) regimen was used for antituberculosis treatment. Pyrazinamide was discontinued 12 months after surgery. The HREZ regimen was continued for 18 to 24 months. The patients were allowed to ambulate with a supportive device 1 week after surgery, and the supportive device should be used for 3 to 6 months. Follow-up radiography was performed to assess internal fixation, bone graft fusion, and tuberculosis recurrence. If necessary, CT or MRI was performed. The ESR, CRP level, and liver and kidney function were monitored. The follow-up was performed over the phone or at outpatient visits.

### Evaluation criteria

#### Clinical parameters

The clinical parameters are as follows: (1) operative time, intraoperative blood loss, postoperative drainage amount, length of hospital stay, and time to bone graft fusion. The VAS was used to evaluate pain, and the ODI was used for function evaluation. (2) The ASIA classification was used to assess neurological function. (3) Laboratory examinations included the ESR and CRP level.

#### Imaging parameters

The imaging parameters include the improvement and loss of the Cobb angle and intervertebral height before surgery, after surgery, and at the final follow-up. The Cobb angle is defined as the intersecting angle between lines vertical to the endplates of the adjacent upper and lower normal vertebrae on the lateral view of a radiograph. Intervertebral height is defined as the vertical height between the upper and lower vertebral bodies of the fused segment on the lateral view of a radiograph. Evidences for bony fusion were defined as the presence of trabecular bone bridging between the bone grafts and the vertebrae on CT sagittal planes. Bone graft fusion was evaluated using the Bridwell criteria [8].

### Statistical analysis

Statistical analyses were performed using SPSS 24.0 for Windows. Data were shown in the form of mean ± standard deviation (SD). With a test level of *α* = 0.05, paired-sample *t* tests were used for comparisons within the groups, and independent-sample *t* tests were used for comparisons between the groups. The difference was considered to be statistically significant when the *P* value was less than 0.05. All *P* values were two-sided.

## Results

### Operative time and the length of hospital stay with the three types of bone grafting

The operative time for bone grafting was 206.1 ± 38.6 min in group A, 240.8 ± 68.4 min in group B, and 238.4 ± 45.0 min in group C. The operative times for bone grafting in group A was significantly shorter than those in groups B and C (*P* < 0.05). No significant differences were found between groups B and C (*P* > 0.05). The length of hospital stay was 18.8 ± 5.1 days in group A, 24.0 ± 12.7 days in group B, and 26.1 ± 9.5 days in group C. The lengths of hospital stay in group A were significantly shorter than those in groups B and C (*P* < 0.05). No significant differences were found between groups B and C (*P* > 0.05).

### Intraoperative blood loss and postoperative drainage amount in the three groups

The intraoperative blood loss was 372.1 ± 150.1 ml in group A, 481.5 ± 241.8 ml in group B, and 603.1 ± 443.5 ml in group C. The intraoperative blood loss in group A was significantly less than that in groups B and C (*P* < 0.05). The postoperative drainage amount was not significantly different between the three groups (*P* > 0.05).

### Pain scores and laboratory parameters in the three groups

The postoperative VAS and ODI scores, ESR, and CRP level were significantly improved compared to those before surgery (*P* < 0.05), although no significant differences were found between the groups (*P* > 0.05).

### Deformity correction, intervertebral height, and loss to the final follow-up in the three groups

No significant differences in the Cobb angle and intervertebral height were found before surgery, after surgery, and at the last follow-up between the four groups (*P* > 0.05).

### Comparison of the time to bone graft fusion in the three groups

The time to bone graft fusion was 7.3 ± 2.1 months in group A, 8.4 ± 3.1 months in group B, and 7.5 ± 3.4 months in group C. No significant differences were found between groups A, B, and C (*P* > 0.05).

### Neurological function recovery

The postoperative neurological function of the patients was improved compared with that before surgery. In the transverse process graft group, neurological function was improved from grade C to grade D in two patients and from grade D to grade E in 14 patients. In the iliac bone graft group, neurological function was improved from grade C to grade D in one patient and from grade D to grade E in seven patients. In the titanium mesh group, neurological function was improved from grade B to grade C in one patient, from grade C to grade D in three patients, and from grade D to grade E in five patients. No significant differences in ASIA classification grades before and after surgery were found between the three groups (*P* > 0.05), but the ASIA grades after surgery were significantly improved compared with those before surgery in the three groups (*P* < 0.05).

### Comparison of complications between the three groups

In the iliac bone graft group, two patients had incisional exudate in the iliac donor site, which healed after several dressing changes, and surgical incision infection was reported in one patient. During the follow-up, the position of the internal fixation was good, and no loosening or breakage complications or cases of tuberculosis recurrence were reported in the patients.

## Discussion

The principles of spinal tuberculosis surgical treatment include thorough lesion removal, relief of spinal cord compression, correction of local segmental angular deformities, and restoration of spinal stability [9]. The stability of the spine is conducive to the relatively stable and static state of local tuberculosis lesions as well as tissue repair and prevention of tuberculosis recurrence [10]. Therefore, bone grafting plays important roles in bone defect repair after the removal of a tuberculosis lesion, correction of kyphosis, and reconstruction of spinal stability. In recent years, posterior approach debridement combined with bone graft and pedicle screw fixation has been used to treat spinal TB. The main advantage of the single posterior approach was that the same incision was used to complete lesion debridement, orthopedic bone grafting, and internal fixation, So we all use this surgical approach [11–14].

As the most widely used bone block graft material, the iliac bone block is the gold standard for bone grafting compared with other graft materials [15, 16]. This graft type has a three-sided cortical bone structure with high mechanical strength and good osteoconductivity and osteoinductivity and is also rich in cancellous bone, which can significantly improve the bone graft fusion rate. From the perspective of biomechanics, Jutte [17] suggested that the bone graft, as a three-sided cortical bone graft, provides good support and is conducive to bone fusion. However, in a study on structural autogenous bone grafting, Tuli [6, 18] suggested that bone fusion occurs only within a depth of a few millimeters of the surface and in the region in close contact with the blood supply in the receiving site, whereas osteonecrosis occurs in the bone graft center. Moreover, the shortcomings of iliac bone grafts include the limited source of the iliac bone, pain in the donor site (more than 6 months of pain and sensory disturbance), and infection.

The porous wall of titanium mesh provides a sufficient contact area between bone powder grafts and the bone bed, allowing tight attachment of the small bone powders in the titanium mesh to the bone to increase the bone graft fusion rate. Moreover, the size and shape of the titanium mesh can be cut arbitrarily, ensuring the area required for bone fusion, correction and prevention of deformities, and prevention of the titanium cage from shifting and dislocation. However, sinking of the titanium mesh into the vertebral body cannot be ignored. Moreover, inserting the titanium mesh is difficult. Sometimes, the implanted bed in the lesioned area must be expanded, slightly compromising spinal stability and significantly increasing the blood loss associated with bone grafting and the time required for grafting [5, 19].

The transverse processes of the thoracic spine protrude to the left and right sides from the junction of the pedicle and the lamina. The transverse process is located between the superior and inferior articular processes and belongs to the posterior column structure. The mature thoracic transverse process is cylindrical and extends to the posterolateral side. It also has a three-sided cortical bone structure, similar to that of the ilium bone and ribs. The harvested transverse process is a ring-shaped cortical bone block that can be trimmed to a suitable size according to the specific shape and length of anterior column bone defects caused by lesion removal. The study by Thanapipatsiri et al. [20] showed that harvesting a transverse process is a safe and feasible procedure without damage to important blood vessels, nerves, muscles, or tendons as long as careful subperiosteal detachment and gentle operative techniques are strictly followed.

According to the anatomy of the transverse process of the thoracic spine, we summarized the requirements for transverse process bone grafting. The study by Panjabi et al. revealed the heights of the vertebral bodies and the lengths of the transverse processes from the three-dimensional quantitative anatomy of the human thoracic vertebrae (T1–T12). In humans, the average length of the transverse process of the thoracic spine is 17.4 mm, the average height of the vertebral body is 14.1–22.7 mm, and the average height of the intervertebral disc is 2–6 mm [21, 22]. These data indicate that in the treatment of single-segment thoracic tuberculosis, the “thick and strong” thoracic transverse process can meet the needs of bone grafting when upper or lower affected vertebral bone destruction does not exceed 1/2 of the height of the vertebral body, or the total height of the damaged upper and lower vertebral bone does not exceed 1/2 of the height of the vertebral body.

The advantages of transverse process grafting are summarized as follows: (1) the transverse process is located in the operative field of posterior debridement of thoracic tuberculosis; therefore, lesion removal, spinal decompression, and spinal deformity correction can be completed using only one incision, which can effectively reduce trauma and bleeding, shorten the operative time, minimize postoperative complications, and thus accelerate postoperative recovery. (2) Transverse process grafting is a simple and convenient method to expose and harvest the transverse process during surgery. The integrity of the ribs, the original attachment structure of the intercostal muscles, and the integrity of the thoracic vertebrae and thorax are all preserved. (3) The transverse process, an autologous bone similar to the ilium and the ribs, has a cortical bone structure. Moreover, when the bone volume of a transverse process is insufficient, bilateral transverse processes can be used for bone grafting. The transverse process can be trimmed according to the specific shape and length of the bone defect to form a columnar cage with cancellous bone at each end such that its size is suitable for the anterior column bone defect caused by lesion removal.

Our studies have shown that the transverse process grafting in the thoracic spine is superior to traditional iliac bone grafting and titanium mesh implantation in terms of operative time, intraoperative blood loss, and length of hospital stay. No differences in VAS and ODI scores, ESRs, CRP levels, the Cobb angle of local segmental kyphosis, or intervertebral height of the surgical site were found between the three groups. Compared with traditional iliac bone grafting and titanium mesh implantation, transverse process grafting can reduce intraoperative trauma and postoperative complications and accelerate postoperative recovery. This new type of bone grafting is associated with minimal surgical trauma and can achieve satisfactory bone fusion, provide good biomechanical strength, and maintain spinal stability.

## Conclusion

Transverse process grafting is a reliable, safe, and effective bone grafting procedure in the treatment of single-segment thoracic tuberculosis. Future research should evaluate the application of thoracic transverse bone grafting in other thoracic single-segment lesions in addition to tuberculosis.

## Data Availability

The datasets used and/or analyzed during the current study are available from the corresponding author on reasonable request.

## Abbreviations

ASIA: American Spinal Injury Association
CRP: C-reactive protein
CT: Computed tomography
E: Ethambutol
ESR: Erythrocyte sedimentation rate
H: Isoniazid
IBG: Iliac bone grafting
MRI: Magnetic resonance
ODI: Oswestry dysfunction index
R: Rifampicin
SD: Standard deviation
TB: Tuberculosis
TBG: Transverse bone grafting
TMG: Titanium mesh grafting
VAS: Visual analog scales
Z: Pyrazinamide
